# Refugee and Asylum-Seeking Children: Interrupted Child Development and Unfulfilled Child Rights

**DOI:** 10.3390/children6110120

**Published:** 2019-10-30

**Authors:** Ziba Vaghri, Zoë Tessier, Christian Whalen

**Affiliations:** 1 School of Public Health and Social Policy, Faculty of Human and Social Development, University of Victoria, Victoria, BC V8P 5C2, Canada; ztessier@uvic.ca; 2 Office of Child and Youth Advocate, Fredericton, NB E3B 5H1, Canada; Christian.Whalen@gnb.ca

**Keywords:** Convention on the Rights of the Child, child rights, refugee, asylum-seeking children, child health, child development, Article 22 of the CRC, children on the move

## Abstract

The 21st century phenomenon of “global displacement” is particularly concerning when it comes to children. Childhood is a critical period of accelerated growth and development. These processes can be negatively affected by the many stressors to which refugee and asylum-seeking children are subjected. The *United Nations Convention on the Rights of the Child* (*CRC*) is the most ratified human rights treaty in history, with 196 States Parties (SPs). The *CRC* provides a framework of 54 articles outlining government responsibilities to ensure the protection, promotion, and fulfillment of rights of all children within their jurisdictions. Among these are the rights of refugee and asylum-seeking children, declared under Article 22 of the *CRC*. Refugee and asylum-seeking children, similarly to all other children, are entitled to their rights under the *CRC* and do not forgo any right by virtue of moving between borders. The hosting governments, as SPs to the *CRC*, are the primary duty bearers to fulfill these rights for the children entering their country. This manuscript provides an overview of the health and developmental ramification of being displaced for refugee and asylum-seeking children. Then, an in-depth analysis of the provisions under Article 22 is presented and the responsibilities of SPs under this article are described. The paper provides some international examples of strengths and shortcomings relating to these responsibilities and closes with a few concluding remarks and recommendations.

## 1. Background and Context

The *United Nations Convention on the Rights of the Child* (*CRC*) is the first human rights treaty that has considered a series of unique human rights specifically for children. The *CRC* has recognized this important phase of life as being a period of accelerated growth and development as well as a time of great vulnerability. Therefore, it has made provisions to promote such development (through, for example, provision and participation rights, such as the right to education and freedom of expression) and to prevent harms to which children may be vulnerable (through protection rights) [1]. The *CRC* is the most highly ratified human rights treaty in history, with ratification from all member States of the United Nations (UN), except the United States of America (United States) [2]. Through ratification, countries become States Parties (SPs) to the *CRC* and have the obligation to (a) harmonize their domestic laws and policies with the *CRC* so that internal systems do not contradict any provision, (b) implement all rights articulated under the *CRC* for children within their jurisdiction, and (c) monitor and report the process of implementation of the *CRC* to a Geneva-based committee known as the United Nations Committee on the Rights of the Child (the Committee hereafter) [1].

This year, the global community is celebrating the 30th anniversary of the *CRC*. Three decades have passed since its adoption by the General Assembly in 1989, and yet, the global community continues to face huge challenges in moving past lip service for children and truly protect their many rights as articulated under the *CRC*. While the unprecedented ratification of this treaty, by all UN Member States but one, speaks strongly about the world’s unanimity for children’s rights, the global community, more often than not, falls short in fulfilling the provisions under the *CRC*. Violations of children’s rights have grave ramifications, particularly when they happen in a systemic manner and on a massive scale. Such is the case in relation to the rights of refugee and asylum-seeking children.

Under Article 22 of the *CRC*, a child or young person who leaves their country of origin to escape war, persecution, or natural disaster, has the right to appropriate protection and provisions, such as health, education, and housing. The rights under the *CRC* govern all children regardless of where in the world they are located; thus, refugee and asylum-seeking children do not lose any of their rights simply because they have moved from one country to another [1]. However, in reality, this is not the case and many of these children, depending on where in the world they move to, are denied many of their rights. Often, the host States, who have a clear set of obligations under the *CRC*, fail to fulfill their responsibilities for refugee and asylum-seeking children and by doing so, subject children to a discriminatory treatment. Such discrimination can not only adversely impact children’s health and development, but also violate their human rights under the *CRC*.

In this manuscript, we will provide an overview of the ramifications of being displaced for children’s health and development and present a critical examination of the rights and provisions under this article. We then outline some international examples of variations in compliance with Article 22 among the host countries, highlighting SPs’ strengths and shortcomings. The manuscript closes with concluding remarks and recommendations for improving the monitoring, reporting, and accountability mechanisms to support refugee and asylum-seeking children. Throughout this manuscript, the term “these children” will refer to children who are “…seeking refugee status or who [are] considered [refugees] in accordance with applicable international or domestic law and procedures, whether unaccompanied or accompanied by [their] parents” under Article 22 of the *CRC*, unless a special sub-category of these children, such as unaccompanied children, are the topic of discussion, in which case they will be identified accordingly [1].

## 2. Refugee and Asylum-Seeking Children

### 2.1. The Scope of the Phenomenon

Globally, millions of individuals are currently displaced and seek refuge for various reasons, such as environmental disasters, economic devastation, violation of human rights, and fear of persecution [3]. In 2018, an estimated 13.6 million individuals were newly displaced. This population consisted of 10.8 million people internally displaced, and 2.8 million refugees and asylum-seekers [4]. Worldwide, the total number of individuals that are forcibly displaced reached 70.8 million in 2018. The majority of refugees globally (67%) originated from only five countries: the Syrian Arab Republic, Afghanistan, South Sudan, Myanmar, and Somalia [4]. However, Venezuelan refugee and asylum-seeker movement within and between borders is increasingly problematic, with an estimated 3.4 million individuals displaced outside the country’s border in 2018, and numbers projected to increase to an estimated 5 million people by the end of 2019. The five countries that host the most refugees are Pakistan, Uganda, Sudan, Germany, and Turkey [4].

Despite the fact that humanitarian assistance mechanisms are in place, the demand far surpasses the supply. Only a small percentage of refugees are resettled each year, and humanitarian visas are difficult to obtain. In 2017, more than 1.19 million people were in need of resettlement; however, only 170,000 were accommodated [5]. Protection of children when they are displaced and on the move is seriously compromised, as there are few legal, safe migration channels accessible. This pressures children and their families to utilize dangerous and risky means for migration, such as paying migrant smuggling services to circumvent restriction and heavily guarded boarders. An estimated 90% of irregular migrants entering Europe in 2015 used such services at some point during their journey [5]. Although less than 3% of the refugee population returned to their countries of origin in 2018, many of them returned to unsafe conditions unsustainable for the protection of child rights [4].

Approximately half of the overall refugee population are children under the age of 18 years [4]. Unaccompanied and separated children are among the most vulnerable of refugees. These children migrate alone or are accompanied by someone other than a parent or legal guardian. Approximately 111,000 unaccompanied children were reported in 2018, although only 27,600 applied for asylum [4]. Due to challenges in reaching and assessing this population, these figures are likely to be an underestimate. The rights declared under the *CRC* for refugee and asylum-seeking children are often violated, resulting in significantly adverse impacts on children’s health and development, which is already in a fragile and compromised state.

### 2.2. Health and Development of Displaced Children

Childhood is a period of rapid growth and development in all physical, mental, spiritual, and social domains [6,7]. Despite their importance, protection and promotion of rights during this stage are seriously compromised when it comes to refugee and asylum-seeking children. Prolonged exposure to unfavourable conditions—for example, hunger, limited access to education and health services, low socioeconomic status, and exposure to violence, war, abuse, and exploitation—has lasting effects on a child’s ability to thrive [6]. These experiences can be considered as Adverse Childhood Experiences (ACEs). The accumulation of ACEs results in increased negative health outcomes and can have lasting impacts later in life. For example, ACEs are associated with mental health problems (e.g., depression, anxiety, and Post-Traumatic Stress Disorder (PTSD)), chronic conditions (e.g., heart disease, diabetes, cancer), high risk-taking behaviours (e.g., substance abuse and unsafe sexual practices), and lower academic achievement and income well into adulthood [8,9,10]. Subsequent lifelong adverse health outcomes in different domains of development, are experienced by children undergoing these circumstances before, during, and after seeking asylum [6].

#### 2.2.1. Physical and Mental Health

The physical health of refugee and asylum-seeking children is negatively affected by living conditions, malnutrition, lack of clean water sources, and limited access to medical services [6]. For instance, there is an increased prevalence of infectious diseases, such as tuberculosis, hepatitis B, HIV, and malaria, as well as higher rates of dental caries and poor oral health, resulting from a lack of health and dental services [6,11,12]. Youth reproductive and sexual health is often overlooked, and access to such education is limited [13]. Refugee children are also more likely to suffer from vaccine-preventable diseases due to the sub-optimal immunization coverage in their country of origin or missing follow-up doses of vaccination while they are on the move [14].

Limited resources in refugee camps can result in maladaptive behaviours such as drug and substance abuse. Such behaviours from adults and children can further exacerbate experiences of sexual abuse and exploitation of children in the form of prostitution, child labour, and domestic servitude [15]. These threats to the protection of children have serious consequences for physical and mental health, and lead to other problematic repercussions, such as unwanted pregnancies, sexually transmitted infections, prolonged and/or more serious maladaptive coping behaviours, increased levels of stress, and physical harm, including the child or young person’s involvement with gangs or criminal activity [16,17].

Trauma is the common experience of all refugee and asylum-seeking children. Many witness the death of a family member or friend or acts of violence to self and others, and experience traumatic events when fleeing their country of origin. Children can also be victims of sexual violence, various types of abuse, neglect, human trafficking, and military recruitment [18]. Damaging personal experiences can traumatize a child and deeply affect their mental health, which can have direct (as is the case with personal injury or death of family members) and indirect (arising from prolonged lack of education, material deprivation) negative impacts on children’s well-being and development [18,19].

Stress and trauma experienced during the risky journey to asylum can later manifest into various long-lasting physical and psychological effects. The presentation of trauma in children can sometimes be difficult to detect but often includes symptoms such as anxiety, mood disorders, depression, sleep disturbances, PTSD, and interpersonal difficulties [20].

Children on the move can also undergo inhumane and harmful detention and can experience lasting emotional and behavioural distress during and after detainment, even if they are housed for a brief period of time [21,22]. Acute as well as chronic stress elevate harmful hormone levels in the body, contribute to chronic health conditions, and are toxic to the developing brain [23]. Many children do not feel safe reporting these predicaments due to a lack of social support and fear that their safety and protection may be further compromised [15].

The trauma of displacement can be compounded by separation from parents or any loss of stable caregivers. Children may be unaccompanied for a number of reasons, such as being sent to claim asylum while the parent stayed behind, separation during the journey, or death of a parent. Neurologically, separation from parents at this critical time can disrupt brain development and impact future physical, emotional, social, and cognitive maturation. Separation, when young, makes children more susceptible to a variety of psychological problems and hinders their ability to make social connections later in life [24]. This prolonged exposure to stress is toxic and can have serious health ramification. Sometimes, the detrimental consequences of such toxic stress can be delayed to later life and manifest themselves in the form of susceptibility to anxiety, mental disorder or predispositions to cancer or other non-communicable diseases [25].

Even when not separated, parents themselves experience trauma and its corresponding consequences as a result of being displaced and on the move. Parents’ suffering from a psychological disorder can also influence the child’s mental, emotional, and behavioural well-being, a phenomenon called transgenerational transmission of trauma [26]. Both the parents’ and child’s exposure to trauma disrupts the dynamic of the child-caregiver dyad, negatively affecting attachment and generating expectations of harm, distrust, and poor emotional and social connectivity [20].

Lastly, the predicament of the young fetus in the womb of the refugee and asylum-seeking mother subjected to all the above-mentioned stressors is of grave significance to child development. While history has documented the horrendous assault to humanity during the Holocaust and the Dutch Famine, retrospective studies of individuals who lived through these events during their intra-uterine lives have illustrated extended consequences of these assaults. The surviving fetuses of such experiences have shown to have higher propensity to develop high blood pressure, diabetes, and obesity and genetic tagging for trauma-related disorders [27,28,29]. A life course approach to child development necessitates awareness of the consequences of traumatic events (e.g., from prolonged periods of displacement and being on the move) and the effects for the developing fetuses of expecting mothers.

#### 2.2.2. Other Social Determinants of Health

Inadequate nutrition and material deprivation are constant components of the predicament of these children. It is important to consider the relationship between socioeconomic status and pre- and post-migration factors. Financial resources affect how efficiently individuals can escape their country of origin, and the food, shelter, and transportation that is accessible during the journey to asylum. Upon arrival, the high-income countries often have the necessary resources to provide refugee children with basic material needs, although other existing challenges affect development and the ability to thrive [22]. For example, unemployment rates are disproportionately higher for refugees than economic migrants or the general population, even years after resettlement. Refugees are also more likely to have temporary jobs, work part-time, and have lower-paying employment [30]. Unfortunately, those who experience barriers in employment have increased challenges in caring for their children, as unemployment frequently leads to material and social deprivation. These conditions extend the period of deprivation and disadvantage for children, and as a result, further adversely impact children’s developmental and health outcomes [31].

#### 2.2.3. Education

Children being displaced and the corresponding loss of education has perhaps the most significant impact when considering human capital. For example, as of 2012, one of the greatest losses in human capital in Syria results from a loss of education, estimated at just under 11 billion US dollars, equivalent to approximately 18% of Syria’s 2010 gross domestic product [32]. The right to education and its importance is emphasised in a number of international treaties, agreements, and universal goals (e.g., the United Nations Sustainable Development Goals and the *CRC*). Although primary education is critical for development, countless children are denied this right in refugee camps or in the cities of the country to which they have fled. About 7.4 million refugee and asylum-seeking children are considered under the United Nations High Commissioner for Refugees (UNHCR) as school aged. In 2017, only 61% and 23% of refugee children were enrolled in primary and secondary education, respectively, compared to 92% and 84% of children globally [33].

Low-income countries have the highest proportions of school-aged refugee and asylum-seeking children who are not in school due to limited resources and a disproportionate influx of these children to their countries compared to developed countries [34]. There are also gender inequities, where girls must overcome significant barriers to access education. Girls are often required to assist with domestic activities, such as collecting water and preparing meals, and must provide care for younger siblings and relatives. Families living in poverty may require daughters to marry young in exchange for goods or to reduce the number of children requiring care, even in the country of resettlement. Sanitation, access to private toilets and menstrual hygiene products, and the stigma around menstruation often forces girls to miss schooling. Overall, significant social and cultural barriers, as well as gender-based discrimination, threaten girls’ ability to fulfil their right to education [35]. Therefore, host countries must ensure that all refugee and asylum-seeking children, regardless of their gender, receive quality education that is taught by a trained teacher following a formal curriculum [34].

Educational delays have lasting consequences on literacy, academic achievement, employment opportunity, and future socioeconomic status [31]. The quality of education is limited by the availability of resources, trained teachers, school materials, and appropriate infrastructure [36]. These limitations also affect a child’s ability to receive secondary and post-secondary education, resulting in only 1% of refugee children going to university compared to 34% of people globally [34]. Benefits from education must be assessed well beyond mere academic achievement. Formal education also provides a sense of normalcy and routine, something that is often lost in the lives of refugee and asylum-seeking children. It can also improve the mental health and development of the child and provide opportunity to contribute to society and achieve goals [22,34]. Women who are educated are better prepared to care for their families, have improved health outcomes, learn independence, have fewer children, and develop leadership skills [33].

Optimal outcomes are experienced when refugee children are included and integrated in the host country’s mainstream education system rather than in segregated learning environments [34]. The right to inclusive education settings guaranteed to disabled children under Article 24 of the *Convention on the Rights of Persons with Disabilities* is extended to all children, including refugee and asylum-seeking children, under the *CRC* through the joint application of Articles 2, 22, 28 and 29 [37,38]. This equal opportunity standard in education places these children on a level playing field with their peers and also accelerates their integration into their host culture [22]. Last but not least, the literature on school and community-based interventions aimed at reducing psychological disorders in refugee and asylum-seeking children indicates that interventions delivered at the school setting can successfully support children in overcoming a great deal of the difficulties associated with forced migration [39].

#### 2.2.4. Acculturation

In their new home, refugee and asylum-seeking children must learn the host country’s language and culture and begin social integration [22]. This adaptation to an unfamiliar culture is gradual and experiences such as marginalization, discrimination, bullying, xenophobia, and acculturation problems are proven harmful for the self-esteem, identity formation, and overall well-being of the child [6,22,40]. Integration into society can be an ongoing process, and participation can be limited by language barriers, cultural differences, and a lack of cultural competencies among professionals and the general public [40].

The *CRC* not only recognizes childhood as a critical state of growth and development, it also underlines the particular vulnerability of refugee children and asylum-seeking children due to all of the shortfalls outlined above. Therefore, to provide special measures of protection and safeguard the development of these children, it puts forth Article 22 and obligates the SPs to uphold these provisions. The following section provides an analysis of this article and presents the main attributes and themes that can assist with monitoring the implementation of Article 22.

## 3. Rights of Refugee and Asylum-Seeking Children under the CRC

Article 22 of the *CRC* obligates all SPs to “… take appropriate measures to ensure that a child who is seeking refugee status or who is considered a refugee in accordance with applicable international or domestic law and procedures shall, whether unaccompanied or accompanied by his or her parents or by any other person, receive appropriate protection and humanitarian assistance in the enjoyment of applicable rights set forth in the present Convention and in other international human rights or humanitarian instruments to which the said States are Parties” (p.6) [1].

The preparation of Article 22, during the course of *CRC* drafting, took place at a time when international law first started to differentiate refugee children from adult refugees [41]. Therefore, the provision under this article captures the broad international consensus that (i) refugee children are owed appropriate protection and international assistance, (ii) all of their rights under the *CRC* as well as other international human rights treaties and humanitarian law must be upheld, (iii) SPs must cooperate with the UN and related agencies in order to protect and assist such children, and (iv) family reunification is a priority obligation of governments serving the best interests of children, having particular regard for unaccompanied and separated children.

### 3.1. The General Principles of the CRC

The principle of non-discrimination (Article 2) and its violations may often be the factor forcing children and their parents to leave their homes in search for safety. Discrimination can happen on a variety of bases, such as ethnicity, religious affiliation, or sexual orientation. Additionally, the mere designation of these children as refugees and asylum-seeking children creates a possible disadvantage and calls for the protection of Article 2 [18]. Within their new home, when these children also belong to groups like minorities, LBGTQ2, or child soldiers, they become further vulnerable. SPs must take positive measures to safeguard de facto equality for children entering their jurisdiction, as well as those children returned to their country of origin [37].

The principle of the best interests of the child (Article 3) should always remain the overarching consideration in implementation of child refugee claims under Article 22 [42]. This principle ought to be respected during all stages of the displacement cycle and decisions at any of these stages, must be appropriately documented through a formal and thorough best interest determination (BID) for each child [18,37,43].

The principle of the child’s right to maximum survival and development (Article 6) and violations to this right are also, more often than not, a root causes of migration [42]. When children leave their country of origin, the protection of child rights becomes even weaker when the journeys become life threatening and high risk [41,44,45]. The SPs are obligated to take special measures to protect children by any means, for instance, through immigration policies facilitating and regulating mobility rights and not repressive detention and deportation practices, in order to protect Article 6 rights [37]. The child’s right to optimal development also must inform Article 22 rights in relation to the immigration policies on the deportation or detention of a child’s parent and/or guardian [37].

Lastly, under the principle of respect for the views of the child (Article 12), SPs must ensure child participation in immigration matters affecting both children and their parents. The child’s best interests will play a role in both instances and children often have “their own migration projects and migration-driving factors” (pp. 9–10) [37]. Children should not be perceived as mere dependents of adult refugees and asylum-seekers; their right under Article 12 must be upheld, ensuring that their views can be expressed freely and given due consideration in relation to their age and maturity. The Committee in its General Comment (GC) No. 22 (the joint GC with the Committee on the Protection of the Rights of All Migrant Workers and Members of Their Families (CMW)) strongly reinforces these as States’ Obligations [37].

The relevance of these four principles to Article 22 becomes clearer when the article is unpacked into its main attributes.

### 3.2. The Main Attributes of Article 22

A number of attributes were identified for each substantive right of the child under the *CRC* [46,47]. These attributes were determined through an exhaustive and critical appraisal of the legal standards within the major guiding documents of the *CRC* (these documents include, but are not limited to, relevant General Comments in the CRC and/or other human rights treaties, relevant articles and provisions under the other human rights instruments, the two *International Covenants on Civil and Political Rights* and on *Economic, Social and Cultural Rights*, relevant sections of Travaux Preparatoires, relevant CRC Concluding Observations, and the UNICEF Implementation Handbook) [47]. The attributes were identified for each *CRC* right in order to make its normative content concrete and to assist with the task of identifying the relevant indicators to monitor that right. In general, through this thorough analysis of relevant documents, the attributes should be able to present the essence and standards of its corresponding right [48]. Four attributes have been identified for Article 22 of the *CRC*, and below is a full discussion of these attributes as a result of the comprehensive analysis and appraisal.

#### 3.2.1. Appropriate Protection and Humanitarian Assistance

Under this article, refugee and asylum-seeking children are neither granted a special status nor any lesser status than children of the host country. They are to be treated as children first and foremost and not as migrants per se, and national immigration policy cannot undermine their rights to education, health, protection, etc., under the *CRC* [42]. The criterion of humanitarian assistance strengthens the notion of “appropriate protection”; for example, these children may require therapy to assist with their recovery from traumatic journeys and successful integration into a new host culture [49,50]. Humanitarian assistance should avoid discriminatory consequences such as differential treatment between categories of entrants in family reunification cases [51], prohibit detention of children and possibly their parents for immigration purposes [18,42], help defend the principle of non-deportation of children [42], and reinforce the child’s right to preserve family life [18,42].

#### 3.2.2. Preservation of Rights

As a general rule, articulated under Article 2, all *CRC* rights apply to all children in every situation, regardless of their background. This general rule governs Article 22 as well, where refugee and asylum-seeking children are entitled to the exact same rights as any other child [1]. Article 22 refrains from reiterating all child rights one by one and rather ensures that refugee and asylum-seeking children preserve both their *CRC* rights and uphold the provisions and protection of other international human rights or humanitarian instruments binding on the relevant SP. Such documents provide much more extensive guidelines for SPs as to how these children are to be protected and describes, in further detail, each right and responsibility. For example, the *Inter-Agency Guiding Principles on Unaccompanied and Separated Children* are especially helpful in clarifying priority focus areas for intervening effectively with this group of particularly vulnerable children, although they should not be interpreted as minimum standards or used to read down any of the rights of unaccompanied minors under the *CRC* [52].

#### 3.2.3. Duty to Protect and Assist through International Cooperation

While governments are the primary duty bearers, all parts of society can play a part in supporting the implementation of children’s Article 22 rights by coming forward. For instance, provision of the right information at the right time can be critical in tracing family members and family reunification. The obligation to protect also contains a clear instruction for all duty bearers to provide children with appropriate due process—processes that do not infringe their rights to be heard and to participate in decision-making that impacts them. Such processes may require providing children with interpreters, a free legal representative, and other means in order to facilitate their meaningful participation. All the processes must be conducted in a child-friendly manner [41]. All professionals involved must be trained in child rights and be familiar with work in culturally sensitive multidisciplinary teams, including psychologists, social workers, and trauma-informed care providers, to name a few [42].

#### 3.2.4. Best Interests and Family Reunification Principles 

Two basic principles should guide every activity related to the refugee and asylum-seeking children: the principle of the best interests of the child and the principle of family unity [41]. After extensive field-testing, UNHCR adopted, in May 2008, its *Guidelines on Determining the Best Interests of the Child* [43].

As for family reunification, it should be based on a robust assessment that upholds the child’s best interest as the primary consideration. Family reunification should not be delayed because of a BID procedure; however, it also cannot trump the child’s best interests and would minimally require a sustainable reintegration plan avoiding any harm to the child in the country of reunification (the non-refoulement principle) and insisting upon the child’s opportunity to participate in the process [18,43]. BID procedure requires a holistic child rights-based approach that considers human and financial resources, training in children’s rights and inter-institutional coordination, drawing upon the cooperation and evidence available from countries of origin, transit, and destination [43].

## 4. Meeting the Provisions of Article 22

In relation to the four attributes of Article 22 described above, in general, countries are struggling to mitigate the effects of displacement and are failing to uphold their international commitments under Article 22 of the *CRC*. Within the context of developed countries, tracking these children is still a challenge, not due to the lack of resources but as a result of inadequate data collection systems that hinder the task of monitoring [53]. The section below presents some examples of SPs’ efforts (or lack thereof) in meeting the provisions of Article 22 and how some States mitigate various challenges, reviewed under all four attributes discussed above.

### 4.1. Appropriate Protection and Humanitarian Assistance

According to this attribute, refugee and asylum-seeking children are not to be granted a special status, nor to be treated as having any lesser status than children of the host country. Therefore, children, regardless of their migration status should continue to realise all their *CRC* rights, including non-discriminatory access to early childhood education, formal and non-formal learning settings, and vocational and technical training. Countries such as Lebanon, Cameroon, and Uganda have strongly established inclusion of refugees in their national school systems in camp or community schools [34]. However, many countries are still struggling to uphold these commitments, such as Kenya, Pakistan, and Malaysia, where only half of the refugee population has access to primary education [34].

Many countries have already implemented legislative commitments and incentives that further reinforce provision of these rights. For example, in Canada, according to Section 30(2) of the Immigration and Refugee Protection Act (IRPA), all children who have yet to receive their immigration status, unless a temporary resident is specifically unauthorized, are automatically eligible without a permit to attend primary and secondary State schools [54]. Once the child becomes a permanent resident of Canada, attendance to public schools is mandatory [55]. Turkey also takes a similar approach to integration, despite hosting the highest number of refugees globally, most of whom are from Syria, and more than 1.7 million of whom are children [56]. Approximately 96% of registered refugees under temporary protection live in urban settings and 80% of children attend Turkish public schools. However, there is still a proportion of children not enrolled in public schools, and many secondary level students who drop out [57]. The Turkish government also runs a Conditional Cash Transfer for Education (CCTE) program in which parents receive financial incentives for each child they send to school. A particular focus is on educating girls, where the incentive is higher [56,57,58].

Refugee and asylum-seeking children should be treated first and foremost as children, although many immigration policies undermine their rights. As the *CRC* Committee states, “detention is never in the best interest of the child”; however, over 100 countries are known to detain children on the basis of their migration status [5,18]. Data regarding the exact countries and number of affected children are unknown, although there is an overall lack of child-friendly accommodations for refugee and asylum-seeking children and their families. Due to the lack of harmonized language surrounding policies for detaining children, many countries mislead and mask their regulations. As the Global Detention Project highlights, countries such as France, Poland, and Spain, amongst many others, state that children “accompany” their parents in detention centers, whereas in Canada, children are “housed as guest” with their parents. This ambiguous language around detention limits the child’s ability to be recognized by the law and further prevents them from claiming their *CRC* rights [59].

Some countries experience a huge influx of refugees and asylum-seekers and are unable to uphold their international commitments. For instance, despite Greece’s ratification of the *CRC*, efforts to mitigate the refugee crisis has led to controversial detention practices, many of which are a product of pressure from the European Union (EU). Many children are housed in detention centers or police stations for periods far exceeding the law of a 45-day maximum. Furthermore, many asylum-seekers are trapped in Aegean Island camps where they are subjected to a lack of food, health services, and legal representatives [60]. Turkey continues to serve as a transit country for many unregistered refugee and asylum-seekers looking to seek protection in the EU. However, due to the EU-Turkey safe-third country agreement, Greece refuses to process asylum claims or transfer refugees and asylum-seekers to the mainland, limiting the number of individuals who can enter Europe [57]. Rather, these islands act as secured facilities to process border procedures and send individuals back to Turkey, which can take months to a few years [60].

Children are frequently detained as a step towards deportation, although this often goes against the principles of non-refoulement, non-deportation, and the child’s right to preserve family life [37]. If a child is to be returned, proper reintegration and reunification is essential for the child’s physical and psychosocial recovery; however, such arrangements are seldom assured. In 2016, 85% of unaccompanied asylum-seeking children from Central America were apprehended in Mexico and returned to their country of origin [5].

### 4.2. Preservation of Rights

Unlike the almost unanimous ratification of the *CRC*, other international human rights treaties or humanitarian instruments do not have a high degree of ratification and support from UN member states. Many countries have yet to ratify the *1951 Convention Relating to the Status of Refugees* (Refugee Convention) or the *1967 Protocol Relation to the Status of Refugees*, the vital conventions that grant refugee and asylum-seekers necessary protection [61]. For example, only 146 SPs have ratified the Refugee Convention, where countries such as India, Bangladesh, Libya, Jordan, Nepal, Lebanon, Saudi Arabia (some of which are hosts of refugees and asylum-seekers) are among the non-ratifying countries. Conventions such as these provide much more in-depth provision for the rights of refugee and asylum-seekers and go into further details about SP responsibilities than what is described in Article 22 of the CRC [61].

Other strategies and frameworks have been adopted to strengthen the multilateralism of SPs and mitigate the increased migration between borders. Although it is not legally binding, 181 countries voted in favour (Hungary and the United States opposed the compact, and Dominican Republic, Eritrea, and Libya abstained their vote) of the *2018 Global Compact on Refugees* (compact) [62]. The *2016 New York Declaration for Refugees and Migrants* also received unprecedented support, where 193 SPs of the United Nations agreed that countries need to provide shelter and care in a more equitable, predictable, and responsible manner to refugees and asylum-seekers [63]. This demonstrates the outstanding support and intention of the global community to work collaboratively to protect the health and well-being of this vulnerable and growing population [62]. The desire to preserve rights is evident, although significant action is needed to keep SPs accountable and to uphold commitments made under international treaties, including the *CRC*.

### 4.3. Duty to Protect and Assist through International Cooperation

In general, SPs are struggling to manage safe migration of children due to insufficient resources, capacity, and political will. Lengthy and difficult processes for rendering appropriate permits, visas, and documentation, and bans on migration create significant barriers that put children in danger [5]. Professionals who interact with refugee and asylum-seeking children need to be trained in child rights, but this is rarely the case.

Research indicates that even in countries such as Denmark, Finland, Iceland, Norway, and Sweden, many border guards and police officers do not have the necessary competencies to deal with these children in a rights-based manner. There is an imbalance in the ways in which refugee and asylum-seeking children’s rights are upheld and a lower standard is tolerated compared to the host country’s children [64]. Often, interpreters and border security officials are not trained in child-friendly communication, which negatively influences initial contact of the children with State representatives. Finland, Norway, and Sweden provide State officials with training for identifying children who may be victims of trafficking, and protocols require the immediate contact of child protection services for unaccompanied refugee and asylum-seeking children [64].

On the positive side, the Nordic countries’ child protection services adopted an interdisciplinary child-friendly approach that aims to reduce the number of interviews and conducts them in an age-appropriate manner. Information is shared among relevant central governing bodies and responsibilities are then allocated to local authorities. This has been proven to reduce levels of anxiety and mitigate the effects of trauma. However, there are many reported cases of long processing periods and miscommunication between agencies which result in a lack of services and care for children [64].

Despite efforts to provide child-friendly services and access to legal support, many children in Denmark report having inadequate information for decision-making, a problem that is particularly problematic for unaccompanied children [65]. Unaccompanied children in Sweden, Finland, and Norway have access to a guardian that represents and supports them during the legal process, although there are variations in the quality of representatives and there are often few complaint mechanisms in place for children [64,66,67].

### 4.4. Best Interests and Family Reunification Principles

An alarming number of refugee and asylum-seeking children leave home unaccompanied by their parents or guardians or are separated from them during the period of being on the move. There are certain regions with a disproportionate number of unaccompanied children traveling alone, such as through the Central Mediterranean route. This route is not only one of the most dangerous and deadly migration channels (1 in 40 rate of mortality) but it is also a highly common route used for unaccompanied and separated children. In 2017, unaccompanied children made up 92% of all children arriving by sea [5]. Other regions such as the United States and Mexico border are also seeing increases in such trends, whereby 100,000 unaccompanied and separated children were apprehended in 2015–2016 [5].

Most countries contain a number of different legal barriers within their policies and procedures that create difficulties for family reunification and thus “prevent many children from enjoying the right to respect and enjoyment of the family life to which they are entitled” (p. 33) [68]. For instance, in certain circumstances, parents who migrated before their children have limited legal means for reunification. Long waiting periods lasting two to three years, insufficient income, age limitations, and restrictions for de facto dependent reunification all prolong periods of separation from loved ones [68]. In the United States for example, “children granted the Special Immigration Juvenile Status, a visa created to provide a permanent legal status for children found to have been ‘abused, abandoned or neglected’ can never exercise family reunion rights” (p.5) [69]. In order to be reunited with family, the child must reach adulthood and prove that their parents are dependent on them [69]. 

There are also significant economic limitations that create barriers for family reunification. Canada and Turkey both have shown good faith to the principle of family reunification; however, even in these countries, the applicants must satisfy a number of criteria to be eligible. For instance, the family member seeking reunification must be able to sponsor the spouse and/or dependent child(ren), demonstrate that they are financially capable of supporting their loved one(s) in the host country, and satisfy regulations of self-sufficiency [70,71]. Alternatively, in Canada, private sponsorship programs are available to help support reunification, although this is reliant on a Canadian citizen or group to fully financially support the individual or family moving to Canada [72]. Furthermore, the initial application claim must indicate all dependents to be eligible for reunification. This creates barriers as many may not be able to meet these requirements or may only be able to sponsor a few family members. Consequently, a spouse and/or the remaining children may be left behind, waiting the lengthy administrative process for the decision of their claim, or until the family is financially capable to support them. Countries can also create barriers by declaring a country in which a family member resides as ‘safe’, deeming them ineligible to apply for asylum and requiring them to follow the lengthy immigration processes instead [5].

There are, however, programs for family reunification that assist vulnerable families fleeing their country of origin. For example, the International Organization for Migration’s Family Assistance Program provides reunification services for individuals with a protection status in Germany. There are service centers in ten countries, including Turkey, Lebanon, and Iraq, that provide safe reunification services, facilitate visa processing and meetings with the countries’ Consulates, and empower families through preservation of rights. Services are provided in their preferred language, and facilities are accessible for individuals with disabilities, as well as designed to be family and child-friendly [73]. For the Canadian resettlement sponsorship applications, determining whether a young child “for whom the family has been caring and whose parents have been killed or are missing” can be considered a de facto dependent and aligns with *CRC* principles. All decisions must be conducted with the child’s best interest in mind and officials must ensure there are no disputes with guardianship. Before finalizing any cases, Canadian officers must ensure that the UNHCR conducted a BID assessment for the child’s referral [74].

## 5. Conclusions

This paper presents just a glimpse into the current global climate for refugee and asylum-seeking children. One area of these children’s lives that is increasingly and rightfully gaining attention is ensuring that children are receiving quality and equitable opportunities for education. There is also more support and acknowledgement from the global community expressing the urgency and commitment to protect child rights and improve the overall conditions of these children, as indicated, for example, by the *Global Compact on Refugees* and the *New York Declaration for Refugees and Migrants* [62,63]. However, it is time to move past expressing the desire to support these individuals and rather work towards implementing the necessary structural and procedural commitments to ensure rights are being preserved.

There is still much to learn about safeguarding migration channels and ensuring that risks are mitigated as children move across borders. Many of the detrimental ramifications on health and development, such as trauma, being separated from loved ones, and exploitation, are exacerbated by the dangerous journeys to safety. At the landing point, globally, there is a need for more child-friendly accommodations and services, especially for children waiting for claims to be processed. During this period of uncertainty and fear, many children are housed with parents in adult facilities and are prevented from claiming other *CRC* rights, such as education, health, and play. In particular, during these periods, unaccompanied refugee and asylum-seeking children need access to all the information that concerns them and must be provided appropriate legal representation, although we see that this is often not the case in areas such as Central America [5]. As discussed above, there are many existing barriers preventing expedited family reunification processes, further prolonging periods of separation and affecting all domains of health.

At a national level, there needs to be better accountability, monitoring, and reporting mechanisms in place to ensure that SPs are upholding their *CRC* and other international commitments.

Collecting timely and accurate data regarding the provision of rights for refugee and asylum-seeking children is necessary to ensure that SPs are accountable for maintaining both their national and international obligations under the *CRC*. The SPs, in accordance with their obligations under the *CRC* in general (and Article 22 in particular) must implement structural commitments in the form of legislation and policies in support of the refugee and asylum-seeking children they host. They must make every effort to ensure that processes align with the general principles of promoting the rights of these children to life and maximum development (Article 6) in a non-discriminatory manner (Article 2). The children’s best interest (Article 3) must remain at the center of their decisions while they provide children with ample opportunities to participate, voice their opinions, and to be heard during these decision-making processes (Article 12) [1]. Last but not least, the SPs must collect and periodically report disaggregated data on all the rights of refugee and asylum-seeking children.

Widespread public awareness raising is required to overcome tribulations such as xenophobia and discrimination demonstrated from the citizens which add to the difficulties faced by these children. These perceptions and behaviours of the citizens can often influence the predicament of newcomers adversely, not just by adding to the stressors in the environment but also by influencing State immigration laws and protocols in a negative manner. Consequently, added means for refusing refugees may be implemented, such as building walls, detaining individuals, closing borders, stopping boats from docking in their country, and returning individuals to their unsafe country of origin by force [5].

Pediatricians, educators, social workers, and professionals working for and/or with refugee and asylum-seeking children are best equipped to advocate for their rights. Action in this domain is two-fold: to ensure that services are conducted in a culturally safe and sensitive manner, and to ensure that the voices of these vulnerable populations are heard. By careful monitoring, reporting, and advocating for the needs of children on the move, care providers can provide a stronger voice to this often-silenced population. By partaking in discussions, joining advocacy groups, and creating safe spaces for newly resettled families, care providers have the ability to influence decision-makers and the general population.

All in all, while some countries have not yet had to deal with complex migration issues to the same degree that others have, with climate change exacerbation, increased civil unrest, and the proliferation of channels for illegal transit, the planet will be experiencing a further increase in the phenomenon of children on the move across its four corners. All countries and indeed, human societies, must come together to reduce the burden and hardship of refugee and asylum-seeking children who travel long distances, experience horrifying journeys, and all too often face discouraging outcomes prior to, during, and even after their resettlement. Working with the provisions of Article 22 while maintaining the principles the *CRC* can serve as a guide in this enormous and remarkably important task.
